# Increased transcription of *TSPO*, *HDAC2*, and *HDAC6* in the amygdala of males with alcohol use disorder

**DOI:** 10.1002/brb3.1961

**Published:** 2020-11-20

**Authors:** Luana Martins De Carvalho, Corinde E. Wiers, Hui Sun, Gene‐Jack Wang, Nora D. Volkow

**Affiliations:** ^1^ National Institute on Alcohol Abuse and Alcoholism National Institutes of Health Bethesda MD USA; ^2^ Center for Alcohol Research in Epigenetics, Department of Psychiatry University of Illinois at Chicago Chicago IL USA; ^3^ Department of Psychiatry Perelman School of Medicine University of Pennsylvania Philadelphia PA USA; ^4^ National Institute on Drug Abuse National Institutes of Health Rockville MD USA

**Keywords:** alcohol, epigenetics, neuroinflammation, TSPO

## Abstract

**Introduction:**

Repeated exposure to high doses of alcohol triggers neuroinflammatory processes that contribute to craving and mood dysfunction in alcohol use disorder (AUD). The upregulation of the translocator protein (TSPO) is considered a biomarker of neuroinflammation, and TSPO ligands have been used as neuroimaging biomarkers of neuroinflammation. Epigenetic mechanisms are also implicated in neuroinflammatory responses to alcohol, and elevated expression of HDAC2 and HDAC6 has been reported in the brain of animals exposed to chronic alcohol.

**Methods:**

The present study examined the transcriptional regulation of TSPO, HDAC2, and HDAC6 in human postmortem brain tissue from males previously diagnosed with AUD (*n* = 11) compared to age‐matched nondependent males (*n* = 13) in four brain regions relevant to AUD: prefrontal cortex (PFC), nucleus accumbens (NAc), hippocampus (HPP), and amygdala (AMY).

**Results:**

Translocator protein mRNA levels in AMY and PFC and HDAC2 and HDAC6 mRNA levels in AMY were upregulated in AUD compared to controls. In AMY, TSPO mRNA levels were positively associated with HDAC2 and HDAC6 mRNA levels, suggesting a possible regulation of TSPO by HDAC2 and HDAC6 in this brain region. In contrast, there were no group differences for TSPO, HDAC2, and HDAC6 in NAc and HPP.

**Conclusion:**

Our study is the first to find upregulated TSPO mRNA levels in AMY and PFC in postmortem brains from AUD consistent with neuroinflammation, and in the amygdala, they implicate epigenetic regulation of *TSPO* by HDAC2 and HDAC6.

## Graphical abstract

First study to identify TSPO mRNA levels in the postmortem AUD brain. Upregulation of TSPO, HDAC2, and HDAC6 mRNA levels in the amygdala, and upregulation of TSPO in the prefrontal cortex in AUD compared to controls. Positive association between the TSPO, HDAC2, and HDAC6 mRNA levels in amygdala.

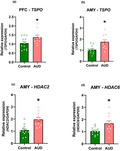

## 1 INTRODUCTION

Alcohol use disorder (AUD) is a chronic relapsing disorder characterized by compulsive drug intake, loss of control in limiting intake, and the development of a negative emotional state when access to the drug is interrupted (Koob & Volkow, 2016). Three distinct stages that involve different brain circuits are implicated in the relapsing curse of AUD (Koob and Volkow, (2010)): The reward circuitry including ventral tegmental area (VTA) and nucleus accumbens (NAcc) plays a key role in the *binge/intoxication* stage; stress circuitry encompassing the extended amygdala (AMY) is involved in the *withdrawal/negative* stage; and a saliency circuitry that includes the prefrontal cortex (PFC), basolateral AMY, hippocampus (HPP), and insula (INS) is involved in the *preoccupation/anticipation* stage (Koob & Volkow, 2016).

Data from postmortem brains of individuals with AUD and from brain of rodents exposed to high doses of alcohol showed evidence that neuroinflammatory process contributed to alcohol‐induced neurotoxicity (Coleman & Crews, 2018; Crews et al., 2017). It has been hypothesized that the increased expression of innate immune signaling molecules and microglial, which are the immune cells of the brain, contributes to the progressive and persistent increase in craving and mood dysfunction in AUD (Crews et al., 2015, 2017). Agonists of Toll‐like receptor 4 (TLR4), which are responsible for activating the innate immune system, increase alcohol self‐administration in mice while knockdown of TLR4 in the central amygdala decreases alcohol self‐administration in alcohol‐preferring rats (Blednov et al., 2011; Liu et al., 2011). Additionally, elevated plasma levels of inflammatory mediators such as TNFα, IL‐1β, IL‐6, and IL‐8 correlated with alcohol craving in individuals with AUD, whereas in IL‐6 null mutant mice alcohol consumption was reduced (Blednov et al., 2012; Heberlein et al., 2014; Roberto et al., 2018).

The translocator protein (TSPO) is a mitochondrial protein that is upregulated in microglia during neuroinflammation (Liu et al., 2014). Ligands to image TSPO with positron emission tomography (PET) have been used as potential biomarkers of neuroinflammation in psychiatric and neurological diseases (Filiou et al., 2017; Rupprecht et al., 2010) including AUD (Hillmer et al., 2017; Kalk et al., 2017; Kim et al., 2018; Lin et al., 2015; Tyler et al., 2019; Wiers et al., 2019). Using in vitro autoradiography, we recently found elevated binding of two frequently used TSPO ligands [^3^H]PK11195 and [^3^H]PBR28 in the thalamus and HPP from brains of alcohol‐dependent compared to nondependent rats, consistent with alcohol‐associated elevation of TSPO expression indicative of neuroinflammation (Tyler et al., 2019). In contrast, in these same animals when we studied them in vivo with PET we reported decreased [^11^C]PBR28 binding in the brain of the alcohol‐dependent rats (Tyler et al., 2019). The reduced binding of PET [^11^C]PBR28 in the brain of dependent rats was consistent with the reduced binding of PET TSPO ligands (including [^11^C]PBR28) reported by clinical studies in the brains of individuals with AUD compared to controls (Hillmer et al., 2017; Kalk et al., 2017; Kim et al., 2018). These discrepant results between in vitro and in vivo findings were confounding and could reflect our limited understanding of TSPO including its in vivo regulation (ref). Beyond its role in neuroinflammation, steroid synthesis, and apoptosis, TSPO was also shown to regulate the development of tolerance to ethanol and to mediate the sensitivity to ethanol sedation in Drosophila (Lin et al., 2015). Given the relevance of TSPO in the responses to chronic alcohol exposure, here we aimed to evaluate the transcriptional regulation of TSPO in postmortem tissue of human AUD.

Epigenetic mechanisms have been implicated in the brain's immune responses to chronic alcohol and to other inflammatory insults (Kaminska et al., 2016; Neal & Richardson, 2018; Placek et al., 2019; Yan et al., 2014). Histone deacetylases (HDACs) are enzymes responsible for removing the transcriptionally permissive acetyl groups on histones. HDACs are classified into four different classes two of which are implicated in immune responses: Class I (HDAC 1, 2, 3, and 8) in innate immunity and cytokine production and Class II (HDAC 4, 5, 6, 7, 9, and 10) in adaptive immunity (Daskalaki et al., 2018; Shakespear et al., 2011). HDAC inhibitors reduce neuroinflammatory responses in the brain and activation of microglia in vitro (Kannan et al., 2013; Patnala et al., 2017; Suliman et al., 2012; Xia et al., 2017; Xu et al., 2018). HDAC expression has also been implicated in the negative affective symptoms in AUD (Pandey et al., 2017). In rodents chronically exposed to alcohol, the increase in anxiety‐like behavior during alcohol withdrawal was associated with higher HDAC activity in amygdala (Pandey et al., 2008). Similarly, elevated activity and expression of HDAC 2 and 6 in NAc and central and basolateral amygdala were reported in alcohol‐dependent versus nondependent rats tested during protracted abstinence (Repunte‐Canonigo et al., 2015). Furthermore, higher innate HDAC levels were associated with reduced global and gene‐specific histone acetylation in the amygdala along with decreased expression of synaptic plasticity‐associated genes and with heightened anxiety‐like behavior and excessive alcohol intake (Palmisano & Pandey, 2017).

Considering the evidence for the involvement of TSPO and HDACs in neuroinflammation and in AUD, the present study examined the transcriptional regulation of *TSPO*, *HDAC2,* and *HDAC6* in human postmortem brain tissue from males previously diagnosed with AUD compared to nondependent males in four brain regions involved in the development and maintenance of AUD: PFC, NAc, HPP, and AMY. We hypothesized an upregulation of TSPO, HDAC2, and HDAC6 in AUD compared to controls, indicating elevated neuroinflammation in AUD individuals and also hypothesized that HDAC2 and HDAC6 regulate TSPO changes in AUD.

## 2 METHODS

### 2.1 Subjects

Human postmortem brain tissue was obtained from the New South Wales Tissue Resource Centre (NSWBTRC) at the University of Sydney, Australia. After review and approval by a National Institute on Alcohol Abuse and Alcoholism (NIAAA) Scientific Advisory Board, the project was also reviewed by the National Institutes of Health (NIH) Office of Human Subjects Research Protections and determined exempt from review by the NIH Institutional Review Board. Four brain regions (PFC, NAc, HPP, and AMY) were analyzed from eleven (*n* = 11) males with severe AUD and thirteen (*n* = 13) males’ controls who did not have AUD. Table 1 summarizes demographics and clinical characteristics. All AUD subjects had alcohol in their blood at the time of death. Controls were donors who consumed less than 20 g of absolute alcohol per day (Sutherland et al., 2016).

**Table 1:** Demographic and clinical characteristics of alcohol use disorder (AUD) and control subjects (mean ± *SD*)

Characteristics	AUD (*n* = 11)	Healthy Controls (*n* = 13)	Statistics	*p*‐value
Age, years	50.55 ± 6.07, *n* = 11	49.94 ± 12.37, *n* = 13	t = −0.264, *df* = 22	*p* = .794
BMI	24.64 ± 5.40, *n* = 11	34.62 ± 11.01, *n* = 13	t = 2.73, *df* = 22	*p* = .012
Daily intake (g)	233.27 ± 118.09, *n* = 11	20.98 ± 21.35, *n* = 12	t = −6.13, *df* = 21	*p* = .005
Drinks per week	125.36 ± 89.99, *n* = 11	10.75 ± 10.30, *n* = 12	t = −4.39, *df* = 21	*p* = .006
BAC (g/100dl)	0.197 ± 0.14, *n* = 10	0.002 ± 0.008, *n* = 10	t = −4.12 *df* = 18	*p* = .001
Pack‐years cigarettes	45.09 ± 19.22, *n* = 11	5.08 ± 15.47, *n* = 12	t = −5.52, *df* = 21	*p* < .001
PMI (hours)	38.91 ± 3.82, *n* = 11	28.85 ± 3.05, *n* = 13	t = −2.08, *df* = 22	*p* = .572
Brain weight (g)	1,387.73 ± 127.7, *n* = 11	1,495.08 ± 95.65, *n* = 13	t = 2.35, *df* = 22	*p* = .028
Age onset drinking	18.55 ± 4.13, *n* = 11	24.0 ± 4.64, *n* = 12	t = 3.23, *df* = 21	*p* = .007

Abbreviations: BAC, blood alcohol concentration; BMI, body mass index; PMI, postmortem interval.

### 2.2 Clinical Assessment and behavioral measures

Clinical characteristics of AUD and control subjects were retrospectively assessed through extensive review of all available medical files followed by a confirmation through donor history questionnaires from the donor's next of kin. Clinical characterization of alcohol use was based on Diagnostic Criteria for Alcohol‐Related Disorders—Alcohol Dependence (DSM‐IV). Alcohol use disorders identification test (AUDIT) was used to assess alcohol consumption, drinking behaviors, and alcohol‐related problems. The number of standard drinks per week and per day was calculated where an Australian standard drink contains 10 grams of alcohol. Quantity and frequency of smoking and pack‐years of smoking were also retrospectively assessed. All details about how NSWBTRC collect demographic, social, medical, pathological, cognitive, psychiatric, medications, and lifestyle factors data are published (Sutherland et al., 2016).

### 2.3 RNA extraction, reversal transcription, and real‐time PCR (qPCR)

Total mRNA was extracted from NAc (*n* = 9 AUD, and *n* = 12 controls, due to restricted tissue availability), PFC (Brodmann areas 8 and 9), HPP and AMY (*n* = 11 AUD and *n* = 13 controls) using the RNeasy Lipid Tissue mini Kit (Qiagen) in accordance with the manufacturer's instructions. Samples were quantified using an Agilent 2100 Bioanalyzer and an RNA 6000 Nano kit. RNA quality for NAc and nanodrop measures for all brain areas are provided in Table S1. RNA samples were stored at −80°C. For each sample, 1 μg of total RNA was used to make complementary DNA (cDNA) using SuperScript^®^ III First‐Strand Synthesis SuperMix for qRT‐PCR kit (Invitrogen) in accordance with the manufacturer's instructions.

The expression levels of target genes were measured using ViiA™ 7 Real‐Time PCR System (Thermo Fisher). The following TaqMan Gene Expression Assays were used: Translocator protein (*TSPO*) Hs00559362_m1, Histone deacetylase 1 (*HDAC 1*) Hs02621185_s1, and Histone deacetylase 6 (*HDAC 6*) Hs00997427_m. The real‐time PCR reactions for each gene were performed using 10 µl of TaqMan™ Universal PCR Master Mix (Thermo Fisher), 0.5 µl of TaqMan assay, and 3.5 µl of ultrapure water. In all reactions, a negative control without cDNA template was tested, and the final reaction volume was kept at 10 µl. The relative quantities of the transcripts were calculated by the delta–delta Ct method (Livak & Schmittgen, 2001) using the GADPH gene as an endogenous control. Table S2 shows the average and standard deviation for the cycle threshold (Ct) levels for GAPDH, TSPO, HDAC2, and HDAC6 in all four brain regions analyzed.

### 2.4 Statistical analysis

Data were analyzed for Gaussian distribution using the Shapiro–Wilk normality test; then, differences in gene expression between AUD and controls were analyzed using the Mann–Whitney test for NAc and unpaired *t* tests for PFC, HPP, and AMY. Our Bonferroni‐corrected alpha for 12 comparisons was 0.004 (0.05/12). We performed exploratory zero‐order Pearson's correlations between gene expression in brain regions where group differences were found and the following variables: age, BMI, brain weight, brain volume, postmortem interval, age of onset of drinking, daily alcohol intake (grams), drinks per week, total drinking (grams), and pack‐years of smoking (number of packs per day x number of years of smoking that number of packs per day) for each group separately. Data were reported as mean and standard deviations. Tests were performed using the statistical analysis package GraphPad Prism version 7.01 and BM SPSS Statistics version 26.

## 3 RESULTS

Table 1 summarizes demographic and clinical characteristics of subjects. Groups did not differ in age (AUD: 50.55 ± 6.07 vs. Controls: 49.94 ± 12.37 years, *p* = .794). AUD compared to control subjects had lower BMI (AUD: 24.64 ± 5.40 vs. Controls: 34.62 ± 11.01 g/m^2^, *p* = .012), started drinking at a younger age (AUD: 18.55 ± 4.13 vs. Controls: 24.0 ± 4.64 years, *p* = .007), had higher daily alcohol intake (AUD: 233.27 ± Controls: 118.09 vs. 20.98 ± 21.35 grams *p* < .001), and greater numbers of drinks per week (AUD: 125.36 ± 89.99 vs. Controls: 0.75 ± 10.30 drinks, *p* < .001). AUD also showed higher blood alcohol concentrations (BAC) at time of death (AUD: 0.197 ± 0.14 vs. Controls: 0.002 ± 0.008 g/100 ml, *p* = .001), with blood alcohol detected in 11 AUD individuals, 6 of whom were reported to be in alcohol withdrawal at the time of death. Additionally, AUD subjects had higher pack‐years cigarettes (AUD: 45.09 ± 19.22 vs. Controls: 5.08 ± 15.47 packs, *p* < .001) and smaller brain weights (AUD: 1,387.73 ± 127.7 vs. Controls: 1,495.08 ± 95.65 grams, *p* = .028) than controls. Postmortem intervals did not differ between groups (AUD: 38.91 ± 12.69 vs. Controls: 28.85 ± 10.99, *p* = .572).

**Figure 1:**
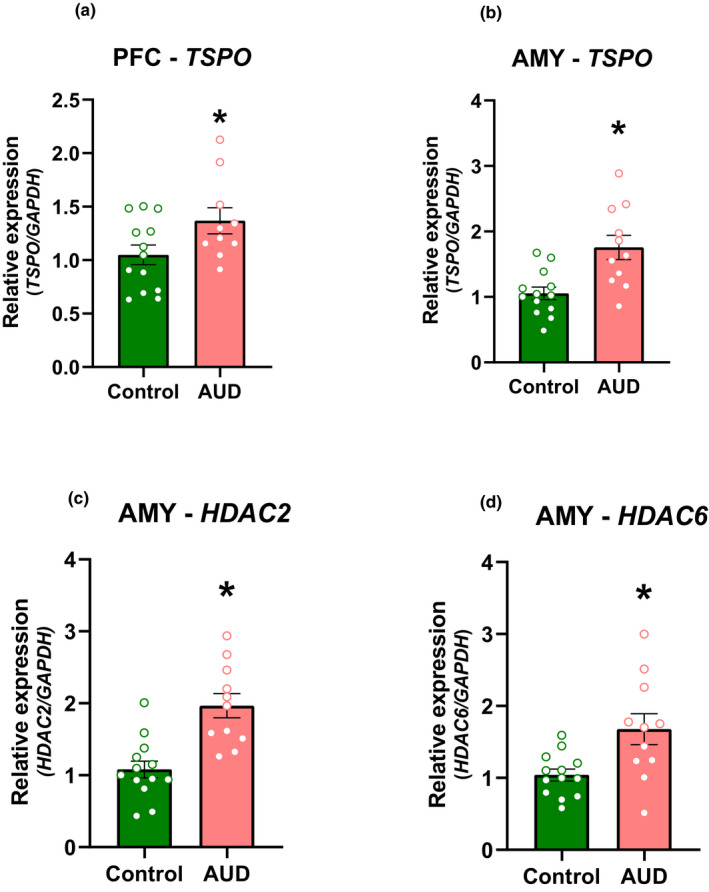
Relative mRNA quantification in the PFC and AMY for AUD and control subjects. Relative mRNA levels of (A and B) TSPO, (C) HDAC2, and (D) HDAC6. In A, B, C, and D *p < .05 different from controls. Unpaired *t* tests were used to compare differences between groups. Results are expressed as mean ± *SEM*. AMY, amygdala; PFC, prefrontal cortex

We compared mRNA levels of *TSPO*, *HDAC2,* and *HDAC6* between AUD and controls in four brain regions: PFC, NAc, HPP, and AMY. One AUD subject was removed from the TSPO mRNA level in PFC, because it was identified as a statistical outlier using the ROUT method (Q = 1%). *TSPO* mRNA was higher in AUD compared to controls in PFC (t = 2.137, *df* = 21, *p* = .0432) and AMY (t = 3.541, *df* = 22, *p* = .0018) (Table 2; Figure 1a and b), and AMY findings remained significant after Bonferroni correction for 12 comparisons. Similarly, *HDAC2* (t = 4.419, *df* = 22, *p* = .0002) and *HDAC6* (t = 2.954, *df* = 22, *p* = .0073) were higher in AMY in AUD compared to controls (Table 2; Figure 1c and d); and *HDAC2* findings remained significant after Bonferroni correction. There were no group differences for *TSPO* in NAc and HPP, and no differences for *HDAC2* and *HDAC6* in PFC, NAc, and HPP (Table 2).

**Table 2:** Relative mRNA level by brain region in alcohol use disorder (AUD) subjects versus controls

mRNA	Region	mRNA levels (mean ± SER)		*p*‐value
		Control	AUD	
HDAC2	PFC	1.054 ± 0.098, *n* = 13	1.353 ± 0.236, *n* = 11	t = 1.233, *df* = 22, *p* = .230
HDAC6	PFC	1.016 ± 0.050, *n* = 13	1.045 ± 0.074, *n* = 11	t = 0.328, *df* = 22, *p* = .745
TSPO	PFC	1.049 ± 0.09, *n* = 13	1.368 ± 0.125, *n* = 10	t = 2.137, *df* = 21, *p* = .043
HDAC2	HPP	1.078 ± 0.114, *n* = 13	0.920 ± 0.098, *n* = 11	t = 1.018, *df* = 22, *p* = .319
HDAC6	HPP	1.139 ± 0.162, *n* = 13	0.984 ± 0.106, *n* = 11	t = 0.767, *df* = 22, *p* = .4511
TSPO	HPP	1.094 ± 0.128, *n* = 13	0.972 ± 0.053, *n* = 11	t = 0.819, *df* = 22, *p* = .421
HDAC2	NAc	1.023, *n* = 11	1.033, *n* = 8	U = 36, *p* = .544
HDAC6	NAc	1.037, *n* = 12	1.00, *n* = 9	U = 41, *p* = .382
TSPO	NAc	1.058, *n* = 12	1.139, *n* = 9	U = 46, *p* = .601
HDAC2	AMY	1.079 ± 0.117, *n* = 13	1.967 ± 0.169, *n* = 11	t = 4.419, *df* = 22, *p* = .0002
HDAC6	AMY	1.040 ± 0.082, *n* = 13	1.667 ± 0.214, *n* = 11	t = 2.954, *df* = 22, *p* = .0073
TSPO	AMY	1.053 ± 0.095, *n* = 13	1.756 ± 0.184, *n* = 11	t = 3.541, *df* = 22, *p* = .0018

Note

Results are expressed as mean ± *SEM*.

Abbreviations: AMY, amygdala; HPP, hippocampus; NAc, nucleus accumbens; PFC, prefrontal cortex.

Exploratory correlations between mRNA levels and drinking, smoking, and demographics (age and BMI) for the AUD group are shown in Table 3 and for controls in Table 4. The correlation analyses in the AUD group showed that in the AMY, *TSPO* (*r* = 0.690, *p* = .01) and HDAC6 (*r* = 0.766, *p* = .005) mRNA levels correlated positively with age. *HDAC6* mRNA levels also positively correlated with pack‐years of cigarettes (*r* = 0.675, *p* = .022). Correlations with daily alcohol intake, drinks per week or BAC with *HDAC6*, *HDAC2,* and *TSPO* were not significant (*p* > .05).

**Table 3:** Zero‐order correlations between relative mRNA levels and demographic and clinical variables in alcohol use disorder (AUD)

AUD	Age	BMI	Age onset drinking	Daily alcohol intake (g)	Drinks per week	BAC	Pack‐years cigarettes		TSPO mRNA level (PFC)		HDAC2 mRNA level (AMY)		HDAC6 mRNA level (AMY)		TSPO mRNA level (AMY)	
Age	1															
BMI	−0.213															
Age onset drinking	0.390	−0.102														
Daily alcohol intake (g)	0.052	0.698*	0.072													
Drinks per week	0.048	0.720*	−0.069	0.968**												
BAC	0.282	0.322	0.212	0.490	0.327											
Pack‐years cigarettes	0.850**	−0.231	0.257	−0.009	0.038	0.000										
TSPO mRNA (PFC)	0.126	−0.221	−0.008	−0.382	−0.356	−0.108		−0.204		1							
HDAC2 mRNA (AMY)	0.132	0.218	−0.159	0.229	0.269	−0.024		−0.169		0.409		1					
HDAC6 mRNA (AMY)	0.766**	−0.009	0.410	0.376	0.355	0.215		0.675*		−0.083		0.431		1			
TSPO mRNA (AMY)	0.690*	−0.033	0.187	0.107	0.106	0.236		0.329		0.644*		0.667*		0.633*		1	

* Correlation is significant at *p* < .05.

** Correlation is significant at *p* < .01.

**Table 4:** Zero‐order correlations between relative mRNA levels and potential covariates in controls

Controls	Age	BMI	Age onset drinking	Daily alcohol intake (g)	Drinks per week	BAC	Pack‐years cigarettes	TSPO mRNA level (PFC)	HDAC2 mRNA level (AMY)	HDAC6 mRNA level (AMY)	TSPO mRNA level (AMY)
Age	1										
BMI	−0.541*	1									
Age onset drinking	0.033	0.138	1								
Daily alcohol intake (g)	0.212	0.114	0.009	1							
Drinks per week	0.349	−0.011	0.106	0.942**	1						
BAC	−0.015	0.073	0.024	−0.116	−0.03	1					
Pack‐years cigarettes	−0.078	0.252	0.045	−0.111	−0.083	0.963**	1				
TSPO mRNA (PFC)	0.462	−0.208	0.377	−0.190	−0.124	−0.371	−0.399	1			
HDAC2 mRNA (AMY)	0.179	−0.380	0.315	−0.133	−0.022	−0.512	−0.403	0.183	1		
HDAC6 mRNA (AMY)	−0.365	0.062	0.154	0.033	−0.016	−0.334	−0.382	−0.391	0.433	1	
TSPO mRNA (AMY)	−0.035	−0.272	0.327	−0.126	−0.062	−0.545	−0.491	0.050	0.939*	0.693*	1

* Correlation is significant at *p* < .05.

** Correlation is significant at *p* < .01

In the AMY, *TSPO* mRNA levels correlated positively with those of *HDAC2* (*r* = 0.667, *p* = .025) and *HDAC6* (*r* = 0.633, *p* = .036). The correlations between *TSPO* mRNA in PFC, NAc or HIPP and *HDAC2*, *HDAC6* mRNA were not significant. In the control group, neither the correlations between *TSPO* and *HDAC2* or *HDAC6*, nor those with demographic variables, reached significance (Table 4).

## 4 DISCUSSION

Here, we found increases in *TSPO* mRNA levels in AMY and a trend in PFC (uncorrected only), as well as increases in mRNA levels in the AMY for HDAC*2* and a trend in *HDAC6* (uncorrected only) in AUD compared to controls. In the AMY, mRNA *TSPO* levels correlated with those for *HDAC2* and *HDAC6*, which provide preliminary evidence for epigenetic regulation of mRNA *TSPO l*evels by *HDAC2* and *HDAC6*.

Although prior studies have investigated changes in the expression of TSPO in HIV, encephalitis, Alzheimer's disease, multiple sclerosis, and stroke (Cosenza‐Nashat et al., 2009; Gui et al., 2020), no transcriptome studies of the human alcohol‐dependent brain reported find differences in TSPO expression levels (Brenner et al., 2020 May 8; Farris et al., 2015; Kapoor et al., 2019; Ponomarev et al., 2012). This make our work the first to investigate TSPO mRNA levels in postmortem brains from human individuals previously diagnosed with AUD. Regarding the differences for HDACs, among those studies (Brenner et al., 2020 May 8; Farris et al., 2015; Kapoor et al., 2019; Ponomarev et al., 2012), just the work published by Kapoor et al., (2019) reported group differences in the expression of HDAC9 in the PFC of individuals with AUD vs. healthy controls. In Ponomarev et al., (2012), despite their finding of novel markers of chromatin modifications in the central and basolateral nucleus of amygdala, as well as in the superior frontal cortex of alcoholic brain, no differences were reported for any HDAC class.

Our finding of increased mRNA levels of *HDAC2* and *HDAC6* in AMY in AUD is consistent with preclinical findings (Repunte‐Canonigo et al., 2015). The finding that HDAC2 and HDAC6 were uniquely increased in AMY but not in PFC, HIPP, or NAc, suggests that the amygdala might be particularly sensitive to epigenetic regulation by chronic alcohol exposure. The unique sensitivity of AMY is in good agreement with studies showing increases in mRNA levels of Hdac2 and Hdac6 in central and basolateral amygdala, respectively, in dependent compared to nondependent adult males rats (Repunte‐Canonigo et al., 2015). In the amygdala, epigenetic regulation by histone acetylation and deacetylation (Baltan et al., 2011; Broide et al., 2007; Volmar & Wahlestedt, 2015) could underlie neuronal plasticity that drives the negative emotionality in AUD (Robison & Nestler, 2011). Preclinical studies have shown that alcohol exposure triggers epigenetic modifications and changes gene expression in the amygdala that have been implicated in anxiety‐ and dysphoria‐like symptoms in alcohol‐dependent rats (Pandey et al., 2017). Additionally, higher expression of *Hdac2* in the AMY of alcohol‐preferring rats compared to nonalcohol‐preferring rats also implicated it in anxiety‐like and in alcohol‐drinking behaviors (Moonat et al., 2013). Furthermore, in the amygdala, studies in rodents reported that chronic alcohol exposure and withdrawal selectively upregulated *Hdac2* (Bohnsack et al., 2018) and that alcohol withdrawal increased HDAC activity (Pandey et al., 2008). Additionally, HDAC inhibitors have been shown to prevent alcohol withdrawal‐related anxiety in rats and to attenuate withdrawal symptoms and relapse in AUD patients (Brady et al., 2002 Aug 1; Lambie et al., 1980; Longo et al., 2002; Pandey et al., 2008). Since the clinical records reported that 6 of 11 of the AUD individuals whose brain we investigated were undergoing withdrawal at the time of death, it is possible that alcohol withdrawal contributed to the increases in *HDAC2* in AUD.

The increases of *TSPO* in the amygdala also identify it as a brain region that might be particularly sensitive to alcohol's neuroinflammatory effects, and the increases in HDAC2 and HDAC6, which are involved in modulating innate and adaptive immunity, are also support this. Mechanistically, in the first moment the higher levels and the positive correlation between TSPO, HDAC2, and HDAC6 could be unreasonable. However, since the alcohol's neuroinflammatory effects are still in the brain and the TSPO is an important mediator in the neuroinflammatory response, its levels remain higher and the higher levels of HDAC could be an attempt to regulate TSPO expression and the neuroinflammatory response. Relevant to neuroinflammation, HDAC6 forms a complex with STAT3, a signal transducer and transcription activator, which regulates synthesis of IL‐10 by macrophages and dendritic cells (Cheng et al., 2014). Furthermore, a study that investigated the genetic and transcription factors required for *Tspo* expression in murine microglia, revealed that Stat3 binds to the *Tspo* promoter and that RNAi mediated silencing of *Stat3* diminish *Tspo* promoter activity (Rashid et al., 2018). Because these results provide evidence for a central function of HDAC6 as cofactor of specific transcription factors to influence gene promoter activity, we hypothesized that HDAC6 could indirectly regulate Tspo transcription via its interaction with STAT3. This rationale is supported by HDAC6 knockdown triggering a reduction of STAT3 phosphorylation in APC cells, as observed by Cheng et al., (2014). Additionally, considering that under stress conditions, TSPO is upregulated in activated proinflammatory (M1) microglia and that studies suggest that microglial activation in response to alcohol is associated with the anti‐inflammatory (M2) microglia phenotype (Crews et al., 2017); we would predict such a mechanism primarily in microglia, although Tspo is also transcribed in neurons.

Although our data are associative and cannot establish causality between the increased HDAC2 and HDAC6 mRNA levels and the upregulation of TSPO mRNA it serves as preliminary evidence to encourage future studies to investigate the effects of HDAC2 and HDAC6 inhibition in TSPO expression and neuroinflammation in AUD. Interestingly, in vitro studies in both neurons and macrophages have shown that HDAC6 attenuates inflammation by protecting against oxidative stress (Rivieccio et al., 2009; Zhang et al., 2019). To the extent that alcohol metabolism generates reactive oxygen species that contribute to oxidative stress (Wu & Cederbaum, 2003), the *HDAC6* increases we observed in AUD could reflect neuronal or glial protective responses against alcohol‐induced oxidative stress. Note that our study as well and many of the preclinical ones were done in males, and future studies are needed to assess whether they generalize to females with AUD.

Our findings of increases in *TSPO* mRNA levels in are consistent with preclinical findings of microglial activation and neuroinflammation induced by chronic alcohol including in vitro studies of increased binding of TSPO ligands in brain (Liu et al., 2014; Tyler et al., 2019). In contrast, it is opposite to findings from PET neuroimaging studies in AUD participants and in chronic alcohol‐exposed rats (Kim et al., 2018), which reported decreased binding of TSPO ligands in brain (Hillmer et al., 2017; Kalk et al., 2017; Kim et al., 2018). In our clinical studies, reduced [11C]PBR28 binding in the brain of AUD participants was inversely associated with plasma cholesterol levels, which is also a ligand for TSPO and led us to hypothesize that it could reflect increased binding competition by high cholesterol levels in AUD. The current findings of increases in TSPO mRNA are consistent with neuroinflammation in AUD and hence strengthen support that reduced TSPO binding in brain PET studies in AUD and in vivo rodent brains might reflect binding competition with endogenous ligands. In the current study, we did not have TSPO protein expression data and we cannot assume a one to one correspondence between mRNA and protein levels. Nevertheless, here we provide the first evidence for transcriptional regulation of TSPO in AUD from postmortem human brains.

One limitation in our study is the use of GAPDH as a single housekeeping gene. GPADH may display variable expression levels (mRNA) across cell types and disease states (Rydbirk et al., 2016), and both alcohol metabolism and GAPDH play a role in cellular dysfunction under stressful conditions (Ou et al., 2010), which may limit the reliability of GPADH for normalization in this sample. In our sample, we found an effect of group on GPADH expression in AMY, PFC, and HPP, but not NAc (see Table S2), and using GPADH for normalization of TSPO and HDAC expression may thus have been influenced the results. Nevertheless, both TSPO and HDAC6 expression in Amygdala remain significantly different between groups when using uncorrected values, strengthening the validity of our findings. Future studies should include multiple housekeeping genes including HPRT1 and 18S that are more stable and are not altered by alcohol consumption.

## 5 CONCLUSION

The present study showed a transcriptional upregulation of *TSPO* in AMY consistent with neuroinflammation. Additionally, the associated upregulation of TSPO with *HDAC2* and *HDAC6* reinforces the involvement of epigenetic mechanism in the regulation of *TSPO* mRNA levels in the amygdala. Further studies are needed to elucidate this mechanism in the context of AUD.

## 6 CONFLICT OF INTEREST

None.

## AUTHOR CONTRIBUTIONS

LMC and HS performed the qPCR analysis. LMC analyzed the data and drafted the manuscript. CEW, GJW, and NDV provided critical revision of the manuscript for important intellectual content. All authors critically reviewed the content and approved the final version for publication.

### Peer Review

The peer review history for this article is available at https://publons.com/publon/10.1002/brb3.1961.

## Supporting information

Table S1

Table S2

## Data Availability

Data available on request from the authors.

## References

[brb31961-bib-0001] Baltan, S. , Bachleda, A. , Morrison, R. S. , & Murphy, S. P. (2011). Expression of histone deacetylases in cellular compartments of the mouse brain and the effects of ischemia. Translational Stroke Research, 2(3), 411–423. 21966324 10.1007/s12975-011-0087-z PMC3182145

[brb31961-bib-0002] Blednov, Y. A. , Benavidez, J. M. , Geil, C. , Perra, S. , Morikawa, H. , & Harris, R. A. (2011). Activation of inflammatory signaling by lipopolysaccharide produces a prolonged increase of voluntary alcohol intake in mice. Brain, Behavior, and Immunity [Internet]. [cited 2020 Jan 20]; 25(Suppl 1), S92–105. Available from: http://www.ncbi.nlm.nih.gov/pubmed/21266194 10.1016/j.bbi.2011.01.008 PMC3098320 21266194

[brb31961-bib-0003] Blednov, Y. A. , Ponomarev, I. , Geil, C. , Bergeson, S. , Koob, G. F. , & Harris, R. A. (2012). Neuroimmune regulation of alcohol consumption: Behavioral validation of genes obtained from genomic studies. Addiction Biology [Internet]. [cited 2020 Jan 20]; 17(1):108–120. Available from: http://www.ncbi.nlm.nih.gov/pubmed/21309947 21309947 10.1111/j.1369-1600.2010.00284.x PMC3117922

[brb31961-bib-0004] Bohnsack, J. P. , Hughes, B. A. , O’Buckley, T. K. , Edokpolor, K. , Besheer, J. , & Morrow, A. L. (2018). Histone deacetylases mediate GABAA receptor expression, physiology, and behavioral maladaptations in rat models of alcohol dependence. Neuropsychopharmacology [Internet]. [cited 2020 Feb 3]; 43(7):1518–1529. Available from: http://www.ncbi.nlm.nih.gov/pubmed/29520058 29520058 10.1038/s41386-018-0034-8 PMC5983537

[brb31961-bib-0005] Brady, K. T. , Myrick, H. , Henderson, S. , & Coffey, S. F. (2002). The use of divalproex in alcohol relapse prevention: A pilot study. Drug and Alcohol Dependence, 67(3), 323–330. 12127203 10.1016/s0376-8716(02)00105-9

[brb31961-bib-0006] Brenner, E. , Tiwari, G. R. , Kapoor, M. , Liu, Y. , Brock, A. , & Mayfield, R. D. (2020). Single cell transcriptome profiling of the human alcohol‐dependent brain. Human Molecular Genetics [internet]., 29(7), 1144–1153. Available from https://academic.oup.com/hmg/article/29/7/1144/5788428 10.1093/hmg/ddaa038 PMC7206851 32142123

[brb31961-bib-0007] Broide, R. S. , Redwine, J. M. , Aftahi, N. , Young, W. , Bloom, F. E. , & Winrow, C. J. (2007). Distribution of histone deacetylases 1–11 in the rat brain. Journal of Molecular Neuroscience [Internet]. [cited 2020 Feb 1]; 31(1), 47–58. Available from: http://www.ncbi.nlm.nih.gov/pubmed/17416969 17416969 10.1007/BF02686117

[brb31961-bib-0008] Cheng, F. , Lienlaf, M. , Wang, H.‐W. , Perez‐Villarroel, P. , Lee, C. , Woan, K. , … Sotomayor, E. M. (2014). A novel role for histone deacetylase 6 in the regulation of the tolerogenic STAT3/IL‐10 pathway in APCs. Journal of Immunology, 193(6), 2850–2862. 10.4049/jimmunol.1302778 PMC4157123 25108026

[brb31961-bib-0009] Coleman, L. G. , & Crews, F. T. (2018). Innate Immune Signaling and Alcohol Use Disorders. Handbook of experimental pharmacology (vol. 248, pp. 369–396). 10.1007/164_2018_92 29500721 PMC6120815

[brb31961-bib-0010] Cosenza‐Nashat, M. , Zhao, M.‐L. , Suh, H.‐S. , Morgan, J. , Natividad, R. , Morgello, S. , Lee, S. C. (2009). Expression of the translocator protein of 18 kDa by microglia, macrophages and astrocytes based on immunohistochemical localization in abnormal human brain. Neuropathology and Applied Neurobiology [Internet]. [cited 2019 Jun 3]; 35(3), 306–328. Available from: 10.1111/j.1365-2990.2008.01006.x 19077109 PMC2693902

[brb31961-bib-0011] Crews, F. T. , Lawrimore, C. J. , Walter, T. J. , & Coleman, L. G. (2017). The role of neuroimmune signaling in alcoholism. Neuropharmacology [internet], [cited 2019 Jun 3]; 122, 56–73. Available from: http://www.ncbi.nlm.nih.gov/pubmed/28159648 10.1016/j.neuropharm.2017.01.031 PMC5493978 28159648

[brb31961-bib-0012] Crews, F. T. , Sarkar, D. K. , Qin, L. , Zou, J. , Boyadjieva, N. , & Vetreno, R. P. (2015). Neuroimmune Function and the Consequences of Alcohol Exposure. Alcohol Research [Internet]. [cited 2018 Oct 27]; 37(2), 331–341, 344–51. Available from: http://www.ncbi.nlm.nih.gov/pubmed/26695754 26695754 10.35946/arcr.v37.2.15 PMC4590627

[brb31961-bib-0013] Daskalaki, M. G. , Tsatsanis, C. , & Kampranis, S. C. (2018). Histone methylation and acetylation in macrophages as a mechanism for regulation of inflammatory responses. Journal of Cellular Physiology. Wiley‐Liss Inc.; 233, 6495–6507. 29574768 10.1002/jcp.26497 PMC13482559

[brb31961-bib-0014] Farris, S. P. , Arasappan, D. , Hunicke‐Smith, S. , Harris, R. A. , & Mayfield, R. D. (2015). Transcriptome organization for chronic alcohol abuse in human brain. Molecular Psychiatry [Internet]. [cited 2020 Jul 23]; 20(11), 1438–1447. Available from: https://pubmed.ncbi.nlm.nih.gov/25450227/ 25450227 10.1038/mp.2014.159 PMC4452464

[brb31961-bib-0015] Filiou, M. D. , Banati, R. B. , & Graeber, M. B. (2017). The 18‐kDa translocator protein as a CNS drug target: Finding our way through the neuroinflammation fog. CNS & Neurological Disorders ‐ Drug Targets, 16(9), 990–999. 10.2174/1871527316666171004125107 28982340

[brb31961-bib-0016] Gui, Y. , Marks, J. D. , Das, S. , Hyman, B. T. , & Serrano‐Pozo, A. (2020). Characterization of the 18 kDa translocator protein (TSPO) expression in post‐mortem normal and Alzheimer’s disease brains. Brain Pathology, 30(1), 151–164. 31276244 10.1111/bpa.12763 PMC6904423

[brb31961-bib-0017] Heberlein, A. , Käser, M. , Lichtinghagen, R. , Rhein, M. , Lenz, B. , Kornhuber, J. , Bleich, S. , & Hillemacher, T. (2014). TNF‐α and IL‐6 serum levels: Neurobiological markers of alcohol consumption in alcohol‐dependent patients? Alcohol [Internet]. [cited 2020 Jan 20]; 48(7), 671–676. Available from: http://www.ncbi.nlm.nih.gov/pubmed/25262503 25262503 10.1016/j.alcohol.2014.08.003

[brb31961-bib-0018] Hillmer, A. T. , Sandiego, C. M. , Hannestad, J. , Angarita, G. A. , Kumar, A. , & McGovern, E. M. , Huang, Y. , O'Connor, K. C. , Carson, R. E. , O'Malley, S. S. , & Cosgrove, K. P. (2017). In vivo imaging of translocator protein, a marker of activated microglia, in alcohol dependence. Molecular Psychiatry [Internet]. [cited 2019 Jun 3]; 22(12), 1759–1766. Available from: http://www.nature.com/articles/mp201710 28242869 10.1038/mp.2017.10 PMC5573660

[brb31961-bib-0019] Kalk, N. J. , Guo, Q. , Owen, D. , Cherian, R. , Erritzoe, D. , Gilmour, A. , Ribeiro, A. S. , McGonigle, J. , Waldman, A. , Matthews, P. , Cavanagh, J. , McInnes, I. , Dar, K. , Gunn, R. , Rabiner, E. A. , & Lingford‐Hughes, A. R. (2017). Decreased hippocampal translocator protein (18 kDa) expression in alcohol dependence: A [11C]PBR28 PET study. Translational Psychiatry [internet], [cited 2020 Feb 18]; 7(1), e996 Available from: http://www.ncbi.nlm.nih.gov/pubmed/28072413 10.1038/tp.2016.264 PMC5545729 28072413

[brb31961-bib-0020] Kaminska, B. , Mota, M. , & Pizzi, M. (2016). Signal transduction and epigenetic mechanisms in the control of microglia activation during neuroinflammation. Biochimica Et Biophysica Acta ‐ Molecular Basis of Disease, 1862(3), 339–351. 10.1016/j.bbadis.2015.10.026 26524636

[brb31961-bib-0021] Kannan, V. , Brouwer, N. , Hanisch, U.‐K. , Regen, T. , Eggen, B. J. L. , & Boddeke, H. W. G. M. (2013). Histone deacetylase inhibitors suppress immune activation in primary mouse microglia. Journal of Neuroscience Research [Internet]. [cited 2020 Jan 30]; 91(9), 1133–1142. Available from: http://www.ncbi.nlm.nih.gov/pubmed/23686642 23686642 10.1002/jnr.23221

[brb31961-bib-0022] Kapoor, M. , Wang, J. C. , Farris, S. P. , Liu, Y. , McClintick, J. , Gupta, I. , … Goate, A. (2019). Analysis of whole genome‐transcriptomic organization in brain to identify genes associated with alcoholism. Translational Psychiatry [Internet]. [cited 2020 Jul 23]; 9(1), 1–11. Available from: 10.1038/s41398-019-0384-y 30765688 PMC6376002

[brb31961-bib-0023] Kim, S. W. , Wiers, C. E. , Tyler, R. , Shokri‐Kojori, E. , Jang, Y. J. , Zehra, A. , … Volkow, N. D. (2018). Influence of alcoholism and cholesterol on TSPO binding in brain: PET [11C]PBR28 studies in humans and rodents. Neuropsychopharmacology [Internet]. [cited 2019 Jan 3]; 43, 1832–1839. Available from: http://www.nature.com/articles/s41386‐018‐0085‐x 29777199 10.1038/s41386-018-0085-x PMC6046047

[brb31961-bib-0024] Koob, G. F. , & Volkow, N. D. (2010). Neurocircuitry of addiction. Neuropsychopharmacology [internet], 35(1), 217–238. Available from http://www.ncbi.nlm.nih.gov/pubmed/19710631 10.1038/npp.2009.110 PMC2805560 19710631

[brb31961-bib-0025] Koob, G. F. , & Volkow, N. D. (2016). Neurobiology of addiction: A neurocircuitry analysis. Lancet Psychiatry [internet], 3(8), 760–773. Available from https://www.ncbi.nlm.nih.gov/pubmed/27475769 10.1016/S2215-0366(16)00104-8 PMC6135092 27475769

[brb31961-bib-0026] Lambie, D. G. , Johnson, R. H. , Vijayasenan, M. E. , & Whiteside, E. A. (1980). Sodium valproate in the treatment of the alcohol withdrawal syndrome. Australian and New Zealand Journal of Psychiatry, 14(3), 213–215. 10.3109/00048678009159381 6783023

[brb31961-bib-0027] Lin, R. , Rittenhouse, D. , Sweeney, K. , Potluri, P. , & Wallace, D. C. (2015). TSPO, a mitochondrial outer membrane protein, controls ethanol‐related behaviors in drosophila. PLOS Genetics, 11(8), e1005366 10.1371/journal.pgen.1005366 26241038 PMC4524697

[brb31961-bib-0028] Liu, G.‐J. , Middleton, R. J. , Hatty, C. R. , Kam, W.‐W.‐Y. , Chan, R. , Pham, T. , Harrison‐Brown, M. , Dodson, E. , Veale, K. , & Banati, R. B. (2014). The 18 kDa translocator protein, microglia and neuroinflammation. Brain Pathology [Internet]. [cited 2019 Jun 3]; 24(6), 631–653. Available from: http://www.ncbi.nlm.nih.gov/pubmed/25345894 25345894 10.1111/bpa.12196 PMC8029074

[brb31961-bib-0029] Liu, J. , Yang, A. R. , Kelly, T. , Puche, A. , Esoga, C. , June, H. L. , Elnabawi, A. , Merchenthaler, I. , Sieghart, W. , June, H. L. Sr, & Aurelian, L. (2011). Binge alcohol drinking is associated with GABAA alpha2‐regulated Toll‐like receptor 4 (TLR4) expression in the central amygdala. Proceedings of the National Academy of Sciences of the United States of America [Internet]. [cited 2020 Jan 20]; 108(11):4465–70. Available from: http://www.ncbi.nlm.nih.gov/pubmed/21368176 10.1073/pnas.1019020108 PMC3060224 21368176

[brb31961-bib-0030] Livak, K. J. , & Schmittgen, T. D. (2001). Analysis of relative gene expression data using real‐time quantitative PCR and the 2‐ΔΔCT method. Methods [Internet]. [cited 2020 Oct 21]; 25(4), 402–408. Available from: https://pubmed.ncbi.nlm.nih.gov/11846609/ 11846609 10.1006/meth.2001.1262

[brb31961-bib-0031] Longo, L. P. , Campbell, T. , & Hubatch, S. (2002). Divalproex sodium (Depakote) for alcohol withdrawal and relapse prevention. Journal of Addictive Diseases, 21(2), 55–64. 10.1300/J069v21n02_05 11916372

[brb31961-bib-0032] Moonat, S. , Sakharkar, A. J. , Zhang, H. , Tang, L. , & Pandey, S. C. (2013). Aberrant histone deacetylase2‐mediated histone modifications and synaptic plasticity in the amygdala predisposes to anxiety and alcoholism. Biological Psychiatry [Internet]. [cited 2020 Feb 2]; 73(8), 763–773. Available from: http://www.ncbi.nlm.nih.gov/pubmed/23485013 23485013 10.1016/j.biopsych.2013.01.012 PMC3718567

[brb31961-bib-0033] Neal, M. , & Richardson, J. R. (2018). Epigenetic regulation of astrocyte function in neuroinflammation and neurodegeneration. Biochimica Et Biophysica Acta ‐ Molecular Basis of Disease. Elsevier B.V.; 1864, 432–443. 29113750 10.1016/j.bbadis.2017.11.004 PMC5743548

[brb31961-bib-0034] Ou, X. M. , Stockmeier, C. A. , Meltzer, H. Y. , Overholser, J. C. , Jurjus, G. J. , Dieter, L. , … Shih, J. C. (2010). A novel role for glyceraldehyde‐3‐phosphate dehydrogenase and monoamine oxidase B cascade in ethanol‐induced cellular damage. Biological Psychiatry [Internet]. [cited 2020 Aug 10]; 67(9), 855–863. Available from: https://pubmed.ncbi.nlm.nih.gov/20022592/ 20022592 10.1016/j.biopsych.2009.10.032 PMC2854240

[brb31961-bib-0035] Palmisano, M. , & Pandey, S. C. (2017). Epigenetic mechanisms of alcoholism and stress‐related disorders. Alcohol [internet], [cited 2019 Jun 2]; 60, 7–18. Available from: http://www.ncbi.nlm.nih.gov/pubmed/28477725 10.1016/j.alcohol.2017.01.001 PMC5464725 28477725

[brb31961-bib-0036] Pandey, S. C. , Kyzar, E. J. , & Zhang, H. (2017). Epigenetic basis of the dark side of alcohol addiction. Neuropharmacology [internet]., [cited 2019 Jun 2]; 122, 74–84. Available from: https://linkinghub.elsevier.com/retrieve/pii/S0028390817300436 10.1016/j.neuropharm.2017.02.002 PMC5479721 28174112

[brb31961-bib-0037] Pandey, S. C. , Ugale, R. , Zhang, H. , Tang, L. , & Prakash, A. (2008). Brain Chromatin Remodeling: A Novel Mechanism of Alcoholism. The Journal of Neuroscience [Internet]. [cited 2019 Jun 4]; 28(14), 3729–3737. Available from: http://www.ncbi.nlm.nih.gov/pubmed/18385331 18385331 10.1523/JNEUROSCI.5731-07.2008 PMC6671100

[brb31961-bib-0038] Patnala, R. , Arumugam, T. V. , Gupta, N. , & Dheen, S. T. (2017). HDAC inhibitor sodium butyrate‐mediated epigenetic regulation enhances neuroprotective function of microglia during ischemic stroke. Molecular Neurobiology, 54(8), 6391–6411. 27722928 10.1007/s12035-016-0149-z

[brb31961-bib-0039] Placek, K. , Schultze, J. L. , & Aschenbrenner, A. C. (2019). Epigenetic reprogramming of immune cells in injury, repair, and resolution. Journal of Clinical Investigation. American Society for Clinical Investigation, 129, 2994–3005. 10.1172/JCI124619 PMC6668667 31329166

[brb31961-bib-0040] Ponomarev, I. , Wang, S. , Zhang, L. , Harris, R. A. , & Mayfield, R. D. (2012). Gene coexpression networks in human brain identify epigenetic modifications in alcohol dependence. The Journal of Neuroscience [Internet]. [cited 2019 Jun 25]; 32(5):1884–1897. Available from: http://www.jneurosci.org/cgi/doi/10.1523/JNEUROSCI.3136‐11.2012 22302827 10.1523/JNEUROSCI.3136-11.2012 PMC3564514

[brb31961-bib-0041] Rashid, K. , Geissl, L. , Wolf, A. , Karlstetter, M. , & Langmann, T. (2018). Transcriptional regulation of Translocator protein (18 kDa) (TSPO) in microglia requires Pu.1, Ap1 and Sp factors. Biochimica Et Biophysica Acta ‐ Gene Regulatory Mechanisms, 1861(12), 1119–1133. 30412797 10.1016/j.bbagrm.2018.10.018

[brb31961-bib-0042] Repunte‐Canonigo, V. , Shin, W. , Vendruscolo, L. F. , Lefebvre, C. , van der Stap, L. , Kawamura, T. , … Sanna, P. P. (2015). Identifying candidate drivers of alcohol dependence‐induced excessive drinking by assembly and interrogation of brain‐specific regulatory networks. Genome Biology [internet]., [cited 2019 Jun 2]; 16(1), 68 Available from: http://genomebiology.com/2015/16/1/68 10.1186/s13059-015-0593-5 PMC4410476 25886852

[brb31961-bib-0043] Rivieccio, M. A. , Brochier, C. , Willis, D. E. , Walker, B. A. , D’Annibale, M. A. , McLaughlin, K. , … Langley, B. (2009). HDAC6 is a target for protection and regeneration following injury in the nervous system. Proceedings of the National Academy of Sciences of the United States of America [Internet]. [cited 2020 Feb 3]; 106(46), 19599–19604. Available from: http://www.ncbi.nlm.nih.gov/pubmed/19884510 19884510 10.1073/pnas.0907935106 PMC2780768

[brb31961-bib-0044] Roberto, M. , Patel, R. R. , & Bajo, M. (2018). Ethanol and Cytokines in the Central Nervous System. Handbook of experimental pharmacology (vol. 248, pp. 397–431). 10.1007/164_2017_77 29236160 PMC7886012

[brb31961-bib-0045] Robison, A. J. , & Nestler, E. J. (2011). Transcriptional and epigenetic mechanisms of addiction. Nature Reviews Neuroscience [internet], 12(11), 623–637. Available from http://www.ncbi.nlm.nih.gov/pubmed/21989194 10.1038/nrn3111 PMC3272277 21989194

[brb31961-bib-0046] Rupprecht, R. , Papadopoulos, V. , Rammes, G. , Baghai, T. C. , Fan, J. , Akula, N. , Akula, N. , Groyer, G. , Adams, D. , & Schumacher, M. (2010). Translocator protein (18 kDa) (TSPO) as a therapeutic target for neurological and psychiatric disorders. Nature Reviews Drug Discovery. 9, 971–988. 21119734 10.1038/nrd3295

[brb31961-bib-0047] Rydbirk, R. , Folke, J. , Winge, K. , Aznar, S. , Pakkenberg, B. , & Brudek, T. (2016). Assessment of brain reference genes for RT‐qPCR studies in neurodegenerative diseases. Scientific Reports, 6, 37116 10.1038/srep37116 27853238 PMC5112547

[brb31961-bib-0048] Shakespear, M. R. , Halili, M. A. , Irvine, K. M. , Fairlie, D. P. , & Sweet, M. J. (2011). Histone deacetylases as regulators of inflammation and immunity. Trends in Immunology. 32, 335–343. 21570914 10.1016/j.it.2011.04.001

[brb31961-bib-0049] Suliman, B. A. , Xu, D. , Williams, B. R. G. (2012). HDACi: Molecular mechanisms and therapeutic implications in the innate immune system. Immunology and Cell Biology. 90, 23–32. 22083527 10.1038/icb.2011.92

[brb31961-bib-0050] Sutherland, G. T. , Sheedy, D. , Stevens, J. , McCrossin, T. , Smith, C. C. , van Roijen, M. , Kril, J. J. (2016). The NSW brain tissue resource centre: Banking for alcohol and major neuropsychiatric disorders research. Alcohol, 52, 33–39. 27139235 10.1016/j.alcohol.2016.02.005 PMC4855871

[brb31961-bib-0051] Tyler, R. E. , Kim, S. W. , Guo, M. , Jang, Y. J. , Damadzic, R. , Stodden, T. , Vendruscolo, L. F. , Koob, G. F. , Wang, G. J. , Wiers, C. E. , & Volkow, N. D. (2019). Detecting neuroinflammation in the brain following chronic alcohol exposure in rats: A comparison between in vivo and in vitro TSPO radioligand binding. The European Journal of Neuroscience, 50(1), 1831–1842. 10.1111/ejn.14392 30803059 PMC10714130

[brb31961-bib-0052] Volmar, C. H. , & Wahlestedt, C. (2015). Histone deacetylases (HDACs) and brain function. Neuroepigenetics. Elsevier Inc; 1, 20–27.

[brb31961-bib-0053] Wiers, C. E. , Martins De Carvalho, L. , Hodgkinson, C. A. , Schwandt, M. , Kim, S. W. , Diazgranados, N. , … Volkow, N. D. (2019). TSPO polymorphism in individuals with alcohol use disorder: Association with cholesterol levels and withdrawal severity. Addiction Biology, e12838 Advance online publication. 10.1111/adb.12838 31713961 PMC7214120

[brb31961-bib-0054] Wu, D. , & Cederbaum, A. I. (2003). Alcohol, oxidative stress, and free radical damage. Alcohol Research & Health [Internet]. [cited 2018 Dec 19]; 27(4), 277–284. Available from: http://www.ncbi.nlm.nih.gov/pubmed/15540798 15540798 PMC6668865

[brb31961-bib-0055] Xia, M. , Zhao, Q. , Zhang, H. , Chen, Y. , Yuan, Z. , Xu, Y. , Zhang, M. (2017). Proteomic analysis of HDAC3 selective inhibitor in the regulation of inflammatory response of primary microglia. Neural Plasticity, 2017, 6237351 10.1155/2017/6237351 28293439 PMC5331322

[brb31961-bib-0056] Xu, J. , Shi, J. , Zhang, J. , & Zhang, Y. (2018). Vorinostat: A histone deacetylases (HDAC) inhibitor ameliorates traumatic brain injury by inducing iNOS/Nrf2/ARE pathway. Folia Neuropathologica, 56(3), 179–186. 30509039 10.5114/fn.2018.78697

[brb31961-bib-0057] Yan, Q. , Sun, L. , Zhu, Z. , Wang, L. , Li, S. , & Ye, R. D. (2014). Jmjd3‐mediated epigenetic regulation of inflammatory cytokine gene expression in serum amyloid A‐stimulated macrophages. Cell Signaling [Internet]. [cited 2020 Jan 27]; 26 (9), 1783–1791. Available from: http://www.ncbi.nlm.nih.gov/pubmed/24703936 10.1016/j.cellsig.2014.03.025 PMC4104139 24703936

[brb31961-bib-0058] Zhang, W.‐B. , Yang, F. , Wang, Y. , Jiao, F.‐Z. , Zhang, H.‐Y. , Wang, L.‐W. , Gong, Z.‐J. (2019). Inhibition of HDAC6 attenuates LPS‐induced inflammation in macrophages by regulating oxidative stress and suppressing the TLR4‐MAPK/NF‐κB pathways. Biomedicine & Pharmacotherapy [internet], [cited 2020 Feb 3]; 117, 109166 Available from: http://www.ncbi.nlm.nih.gov/pubmed/31255993 10.1016/j.biopha.2019.109166 31255993

