# Supplementary material for: Increased transcription of TSPO, HDAC2, and HDAC6 in the amygdala of males with alcohol use disorder
Source: Brain Behav. 2020 Nov 20;11(2):e01961. doi: 10.1002/brb3.1961 (PMC7882159; doi:10.1002/brb3.1961)
Supplement: Supplementary file 2 — Table S2 [file BRB3-11-e01961-s002.docx]

| Gene | Brain Region | Control Group | AUD group | p-value |
| --- | --- | --- | --- | --- |
| GAPDH | AMY | 20.52 ± 0.83 | 21.53 ± 0.94 | <0.0001* |
| HDAC6 | AMY | 26.85 ± 0.78 | 27.25 ± 0.68 | 0.022* |
| HDAC2 | AMY | 28.18 ± 0.66 | 28.27 ± 0.65 | 0.556 |
| TSPO | AMY | 26.90 ± 0.60 | 27.19 ± 0.64 | 0.049* |
| GAPDH | PFC | 21.43 ± 0.47 | 21.95 ± 0.86 | 0.005* |
| HDAC6 | PFC | 29.33 ± 0.47 | 29.82 ± 0.63 | 0.001* |
| HDAC2 | PFC | 29.64 ± 0.39 | 29.86 ± 0.53 | 0.032* |
| TSPO | PFC | 28.24 ± 0.37 | 28.21 ± 0.56 | 0.984 |
| GAPDH | HPP | 22.47 ± 0.70 | 22.01 ± 0.71 | 0.020* |
| HDAC6 | HPP | 28.91 ± 0.58 | 28.66 ± 0.44 | 0.051 |
| HDAC2 | HPP | 29.23 ± 0.52 | 29.08 ± 0.63 | 0.274 |
| TSPO | HPP | 28.25 ± 0.54 | 27.96 ± 0.62 | 0.056 |
| GAPDH | NAc | 21.52 ± 0.54 | 21.64 ± 0.67 | 0.349 |
| HDAC6 | NAc | 28.08 ± 0.85 | 28.61 ± 1.98 | 0.489 |
| HDAC2 | NAc | 28.44 ± 0.52 | 28.86 ± 1.31 | 0.633 |
| TSPO | NAc | 27.80 ± 0.44 | 27.81 ± 0.67 | 0.8442 |

**Supplementary table 2**. Cycle Threshold (Ct) levels, expressed as mean ± standard deviation, for GAPDH, TSPO, HDAC2 and HDAC2 mRNA by brain region. AMY: Amygdala; PFC: Pre-frontal cortex; Hipp: hippocampus; NAc: Nucleus Accumbens. AUD: Alcohol Use Disorder group.
